# Oral Microbiota Identifies Patients in Early Onset Rheumatoid Arthritis

**DOI:** 10.3390/microorganisms9081657

**Published:** 2021-08-03

**Authors:** Anders Esberg, Linda Johansson, Ingegerd Johansson, Solbritt Rantapää Dahlqvist

**Affiliations:** 1 Department of Odontology, Umeå University, 901 87 Umeå, Sweden; ingegerd.johansson@umu.se; 2 Department of Public Health and Medicine, Rheumatology, Umeå University, 901 87 Umeå, Sweden; linda.e.johansson@umu.se

**Keywords:** rheumatoid arthritis, oral microbiota, periodontitis, 16S rDNA sequencing

## Abstract

Rheumatoid arthritis (RA) is the most common autoimmune inflammatory disease, and single periodontitis-associated bacteria have been suggested in disease manifestation. Here, the oral microbiota was characterized in relation to the early onset of RA (eRA) taking periodontal status into consideration. 16S rRNA gene amplicon sequencing of saliva bacterial DNA from 61 eRA patients without disease-modifying anti-rheumatic drugs and 59 matched controls was performed. Taxonomic classification at 98.5% was conducted against the Human Oral Microbiome Database, microbiota functions were predicted using PICRUSt, and periodontal status linked from the Swedish quality register for clinically assessed caries and periodontitis. The participants were classified into three distinct microbiota-based cluster groups with cluster allocation differences by eRA status. Independently of periodontal status, eRA patients had enriched levels of *Prevotella pleuritidis*, *Treponema denticola*, *Porphyromonas endodontalis* and *Filifactor alocis* species and in the *Porphyromonas* and *Fusobacterium* genera and functions linked to ornithine metabolism, glucosylceramidase, beta-lactamase resistance, biphenyl degradation, fatty acid metabolism and 17-beta-estradiol-17-dehydrogenase metabolism. The results support a deviating oral microbiota composition already in eRA patients compared with healthy controls and highlight a panel of oral bacteria that may be useful in eRA risk assessment in both periodontally healthy and diseased persons.

## 1. Introduction

Rheumatoid arthritis (RA) is the most common autoimmune inflammatory disease [1], affecting nearly 1% of the Caucasian population [2]. The disease is characterized by inflammation of the synovial joints, with subsequent damage of cartilage, bone and/or tendons. The presence of autoantibodies, such as rheumatoid factor (RF) and anti-citrullinated peptide antibodies (ACPAs), are hallmarks of RA [3]. In parallel with the pre-symptomatic evolution of autoantibodies, RA markers have expanded to include circulating pro- and anti-inflammatory cytokines [4,5]. The development of RA involves a concert of host (e.g., genetic, epigenetic factors) and environmental factors (e.g., smoking, infections and stress) with the microbiota, particularly that of the alimentary tract, offering unexplored potential effector mechanisms [1,6,7]. The oral cavity, which is the first part of the alimentary tract, harbors billions of bacteria with more than 700 different identified species [8], of which certain species may trigger RA autoimmunity [6,7,9,10].

Periodontitis, a bacteria-induced inflammatory disease, is associated with manifest RA, possibly through periodontitis-associated bacteria, particularly *Porphyromonas gingivalis* [7,11]. *P. gingivalis* is a Gram-negative anaerobic bacterium with the ability to produce extracellular arginine-gingipain A and B proteases and endogenous peptidylarginine deiminase (PPAD) leading to tissue destructive processes and induction of protein citrullination [12,13]. Citrullination is a post-translation modification that converts the amino acid arginine to amino acid citrulline in certain proteins. These citrullinated proteins are recognized as a foreign antigen by the immune system resulting in the upregulation of inflammatory action such as in RA [14]. However, some studies contradict that *P. gingivalis* PPAD enzymatic activity is a major virulence factor in early-onset RA [15,16]. In addition, the periodontitis-associated *Aggregatibacter actinomycetemcomitans* (*Aa*) and its pore-forming toxin leukotoxin A trigger dysregulated activation of citrullinating enzymes in neutrophils, stimulating hypercitrullination suggesting *Aa* as a candidate-bacterium for autoimmunity in RA [17]. However, the role of *P. gingivalis* and *Aa* remains unclear in RA development. Thus, some studies using 16S ribosomal RNA (16S rRNA) high-throughput sequencing find a higher abundance of *P. gingivalis* and/or *Aa* in early and established RA as well as in patients at risk for RA (ACPA positive arthralgia) [6,9,18], whereas others do not [19,20,21,22]. Anyhow, several of the studies emphasize the importance of characterizing the entire microbiota, map to the species level, and distinguishing between periodontally healthy and diseased states and treatment immune modifying anti-rheumatic drugs.

The aim of the present study was to evaluate the oral microbiota in periodontally defined, untreated early RA using multiplex 16S rRNA gene sequencing and a curated database for species-level classification of oral bacteria.

## 2. Materials and Methods

### 2.1. Study Design

A total of 61 patients with early-onset RA (eRA), i.e., symptom duration ≤12 months and who fulfilled the 1987 American Rheumatism Association criteria for RA [23], were consecutively included in the study together with 59 healthy subjects matched for sex and age. For 2 cases >85 years of age, no controls were identified. Disease activity scores (DAS28) [24], including swollen and tender joint counts, erythrocyte sedimentation rate (mm/h), patient global assessment (visual analogue scale, mm), and also C-reactive protein level (mg/L), were assessed by a rheumatologist at inclusion (index date) [24]. All participants answered a questionnaire on tobacco use. Smoking status was classified as never or ever (current and former smoking) smoker. None of the cases or controls had taken antibiotics during the preceding 3 months. Of the patients, 16 (26%) had corticosteroid treatment (prednisolone, mean dose (SEM) 8.3 (1.6) mg/day) prescribed by the referring general practitioner, the month prior to inclusion, but none had any disease modifying anti-rheumatic (DMARD) treatment or any other known autoimmune condition (e.g., Sjogren’s syndrome)**.** ACPA and RF were analysed as previously described [4]. Participant characteristics are presented in Table 1.

### 2.2. Saliva Sampling

Whole stimulated saliva was collected at the inclusion of cases and controls. Saliva, collected for 3 min into ice-chilled sterile test tubes while participants chewed on a 1 g piece of paraffin wax, was performed at least 3 h after the nearest meal or tooth brushing. All samples were stored at −80 °C. Whole stimulated saliva sample was selected as an oral microbiota source as it has been suggested to detect both tooth microbiota, including periodontal pathogens and bacteria colonising other soft mucosal surfaces of the mouth [25].

### 2.3. DNA Extraction and 16S rRNA Gene Sequencing 

Genomic DNA was extracted from saliva samples and positive and negative controls using the GenElute™ Bacterial Genomic DNA Kit (Sigma-Aldrich, St. Louis, MO, USA) [26]. Briefly, the samples were centrifuged for 5 min at 13,000 rpm, lysed in buffer with lysozyme and mutanolysin, treated with RNase and Proteinase K, and purified and washed. All DNA extractions were performed by the same person and with kits from the same batch. The quality of the extracted DNA was estimated using a NanoDrop 1000 Spectrophotometer (Thermo Fisher Scientific, Uppsala, Sweden) and the quantity by the Qubit 4 Fluorometer (Invitrogen, Thermo Fisher Scientific, Waltham, MA, USA). The mean yield from 200 μL saliva was 32 ng/μL (range 6 to 92 ng/μL) and the ratio of the absorbances at 260 nm and 280 nm was 1.8 or higher. A mixture of known mock bacteria was used as positive control and sterile water as a negative control.

Bacterial 16S rDNA amplicons were generated from the v3-v4 regions by PCR using fusion primers with 341F (ACGGGAGGCAGCAG) forward and 806R (GGACTACHVGGGTWTCTAAT) reverse primers from saliva and the mock community extracted DNA as described by Caporaso [27]. Equimolar libraries were pooled and purified using AMPure XP beads (Beckman Coulter, Stockholm, Sweden) and sequenced using the Illumina Miseq platform. All samples were analysed in the same run.

Obtained sequences were de-multiplexed using deML [28], pair-end reads fused, primers, ambiguous and chimeric sequences removed, and amplicon sequence variants (ASVs) obtained using the open-source software package DADA2 within the QIIME2 microbiome bioinformatics platform (https://qiime2.org, accessed on 1 September 2020) [29,30]. ASVs with >1 read were taxonomically classified against the expanded Human Oral Microbiome Database (eHOMD) (http://www.homd.org, accessed on 1 September 2020) [31]. Only ASVs with at least 98.5% identity with a named species or unnamed phylotype in eHOMD were retained, and those with the same HMT number were aggregated. The negative control contained 34 reads and the mock species were correct with 23 or more reads. Therefore, all comparisons were based on taxa with at least 40 reads. For simplicity, all species levels, i.e., species and phylotypes, were referred to as species in the text.

### 2.4. Assessment of Periodontal Status 

The Swedish quality register on caries and periodontal diseases (SKaPa, www.SKaPa.reg (accessed on 2 July 2021)) compiles and quality controls, information from electronic records from all public dental health care and some private clinics in Sweden. Data on probing pocket depth (PPD) measured at 4 or 6 tooth sites were obtained by linking the RA cases and controls to the SKaPa register using unique personal identification numbers. Data could be retrieved for 48 eRA patients and 30 healthy controls. Data from the visit closest to the index date, i.e., for eRA patients mean (SD) 0.5 (1.8) and controls 0.8 (1.8) years before the index date. PPD, which was used as a proxy for loss of periodontal supportive tissues, was classified as no (<4 mm), mild (4 mm), moderate (5 mm) and severe (>6 mm) loss [32]. For records where 4 tooth sites (mesial, distal, buccal and lingual) were measured, the mesial measurement was used to impute the mesio-lingual and mesio-buccal pockets, and the distal measurement to impute disto-lingual and disto-buccal pockets. For teeth missing due to hypodontia, orthodontic treatment, trauma, caries or failed endodontic treatment, periodontal probing pockets were imputed as <4 mm. Periodontal pockets of teeth missing due to periodontitis or unspecified reasons were imputed using an age- and tooth-based algorithm [33]. Third molars were excluded from evaluation.

### 2.5. Complementary Quantitative PCR

Presence of *Aa* and *P. gingivalis* in saliva was evaluated using LtxA [34] or Pg-F/Pg-R primers [35], respectively, in a PCR mixture of 20 μL containing 10 μL KAPA CYBR FAST (KK4601; Kapa Biosystems, Sigma-Aldrich Merck, Stockholm, Sweden) and 4 μL DNA template.

### 2.6. Statistical Analyses

SPSS (IBM Corp. version 25.0) and PAST 3 [36] software packages were used for descriptive and inferential statistics. Group differences between continuous variables were tested using the Mann–Whitney U test, and categorical variables were tested using the chi-squared test. All tests were two-sided, and correction for multiple comparisons was conducted using the Benjamini–Hochberg false discovery rate (FDR). *p*-values were considered significant at FDR <0.05. Orthogonal partial least square discriminant analysis (OPLS-DA) (SIMCA 15; Sartorius Stedim Data Analytics AB, Malmö, Sweden) was used to search for differences in oral microbiota between the cases and controls. Clustering of subjects by bacterial taxa in the saliva microbiota was performed by agglomerative hierarchical cluster analysis using Ward’s method, and the linear discriminant analysis (LDA) effect size (LEfSe) method was used for high-dimensional class comparisons [37]. Potential molecular functions of the saliva microbiota were predicted using the Phylogenetic Investigation of Communities by Reconstruction of Unobserved States (PICRUSt2) plugin for QIIME2 and converted to functions via the Kyoto Encyclopedia of Genes and Genomes (KEGG) Orthology database (https://www.genome.jp/kegg/ko, accessed 1 September 2020) [38].

## 3. Results

### 3.1. Overall Participant and Microbiota Characteristics 

The eRA patients were dominated by women, and present or past smoking was more prevalent among eRA patients than controls. Further, the eRA patients tended to have fewer teeth and more teeth with a 6 mm PPD than the controls (Table 1).

**Figure 1:**
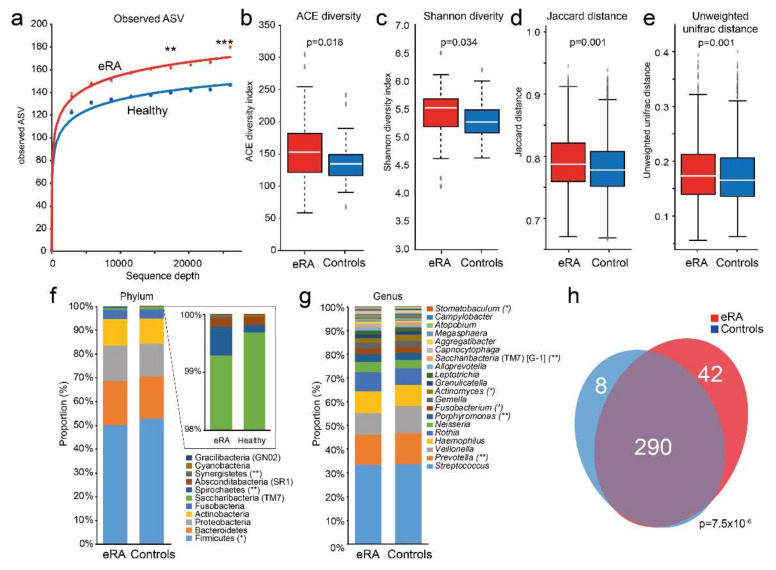
Comparison of saliva microbiota diversity, (dis)similarity, evenness and shared species between early onset RA and healthy subjects. (**a**) Rarefaction curves for amplicon sequence variants (ASV) in early onset RA (eRA) and healthy control subjects. Box plots of (**b**) ACE diversity, (**c**) Shannon diversity, (**d**) Jaccard distance and (**e**) unweighted unifrac distance. Stacked bar graph of top 20 (**f**) phyla and (**g**) genera for eRA patients and healthy control subjects. ***, ** and * indicate <0.001, <0.01 and <0.05, respectively. (**h**) Venn diagram illustrating overlapping and unique species in e RA and healthy controls. A full list of detected phyla, classes, orders, families, genera and species are presented in Table S1.

16S rRNA gene sequencing of saliva DNA from the cases and the controls yielded 15,221,851 reads, which after trimming, denoising and removal of potential chimeric sequences, retained a mean of 31,706 paired-end reads per sample encompassing 2584 ASVs. The mean number of observed ASVs was significantly higher in the saliva microbiota of eRA than in healthy controls (*p* = 0.005 at a sequencing depth of 17,333 reads and *p* < 0.001 at a sequencing depth of 26,000 reads; Figure 1a). The eRA patients had higher alpha diversity indexes (ACE (*p* = 0.018, Figure 1b) and Shannon (*p* = 0.034, Figure 1c)), and beta diversities for categorical estimates (Jaccard (*p* = 0.001, Figure 1d)) and unweighted UniFrac (*p* = 0.001, Figure 1e) than controls.

The 2584 ASVs matched 415 16S rRNA gene sequences in eHOMD with >98.5% sequence identity and with >40 reads, representing 340 species or unnamed phylotypes in 74 genera and 11 phyla. At the phylum level, Firmicutes (50.0%), Bacteriodetes (18.6%), Proteobacteria (15.0%) and Actinobacteria (11.1%) dominated (Figure 1f), and at the genus level *Streptococcus* (33.5%), *Prevotella* (12.9%), *Veillonella* (10.3%), *Haemophilus* (9.0%) and *Rothia* (7.5%) (Figure 1g). The eRA patients had, compared with the controls, significantly lower abundance in phylum Fimicutes and genera *Prevotella* and *Actinomyces*, and higher in phyla Spirochaetes and Synergistetes, and in genera *Porphyromonas* and *Fusobacterium* (Figure 1f,g). The microbiota composition did not differ significantly between sexes (*p* = 0.531) or smoking status among all participants (*p* = 0.314) or within the eRA or control subgroups separately, and not between RF+/− (*p* = 0.583), ACPA+/− (*p* = 0.081) or corticosteroid treated (*p* = 0.403) eRA subgroups (PERMANOVA, 9999 permutations, FDR-adjusted, Euclidean distance).

The dominant species were the *Streptococcus infantis*/*oralis*/*mitis* complex (16.3%), *Haemophilus parainfluenzae* (7.8%), *Streptococcus salivarius* (7.7%), *Rothia mucilaginosa* (6.4%) and *Prevotella melaninogenica* (5.6%) (Table S1). Of the 340 assigned species, 290 were detected in both eRA patients and controls, 42 were unique for eRA patients, and 8 were unique for healthy subjects (*p* = 7.5 × 10^−6^, Figure 1h). All identified species are listed in Table S1.

### 3.2. Oral Microbiota Clustering of Participants 

**Figure 2:**
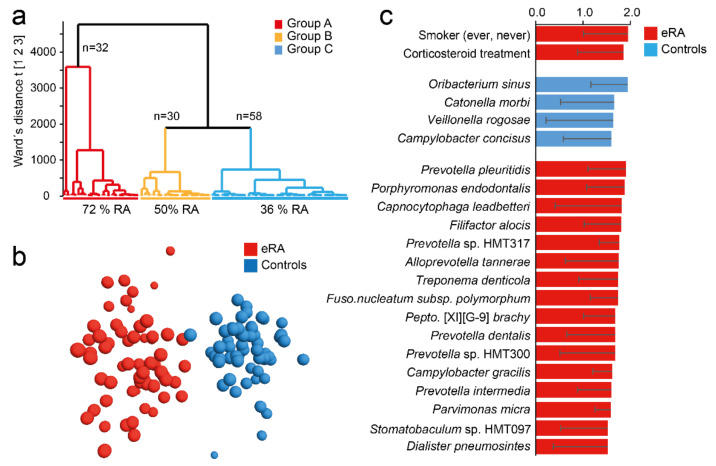
Saliva microbiota hierarchical cluster groups and early onset RA prevalence. (**a**) Dendrogram from hierarchical clustering based on 16S rDNA sequencing defined taxa in saliva samples. (**b**) OPLS-DA loading scatter plot based on a model including early-onset RA (eRA) and healthy subjects (controls) as dependent variable (Y) and abundance of species, phylotypes and genera detected in saliva samples as the independent variable block (X). (**c**) Bar graph showing top 20 bacterial species and smoking status influential in the OPLS-DA model and expressed as Variable Importance in Projection (VIP) values with 95% confidence interval. Red colour represents eRA and blue colour healthy controls. For a full list of VIP values see Table S2.

Following the differences in microbiota richness and diversity between eRA patients and healthy controls, participants were classified by hierarchical clustering based on their microbiota profiles (Figure 2a). This identified three subgroups: group A (*n* = 32) dominated by eRA patients (72%), group C (*n* = 58) by healthy controls (64%) and group B (*n* = 30) with equal proportion of eRA and healthy controls (*p* = 0.0051). As this indicated a systematic difference in saliva microbiota structure in eRA versus healthy control participants, bacterial species that characterized eRA and healthy control subjects were searched by a supervised multivariate OPLS-DA regression. This rendered a model with an explanatory power of 87% (R^2^ = 0.87) and predictive power of 42% (Q^2^ = 0.42) (Figure 2b). The top 20 most influential taxa for the separation of the eRA cases and healthy controls included five species in *Prevotella* (*pleuritidis*, spp. HMT 317 and HMT 300, *dentalis*, and *intermedia*), *Porphyromonas endodontalis*, *Capnocytophaga leadbetteri*, *Filifactor alocis*, *Alloprevotella tannerae*, *Treponema denticola*, *Fusobacterium nucleatum subsp. polymorphum* and more (Figure 2C). Healthy subjects were characterized by abundances of *Oribacterium sinus*, *Catonella morbi*, *Veillonella rogosae* and *Campylobacter concisus* (Figure 2c). A full list of species in the model are shown in Table S2.

Considering *Aa* and *P. gingivalis*, totally 5 subjects (4.2%) all being eRA patients, harboured *Aa* and a total of 21 subjects (17.5%) harboured *P. gingivalis* with a 1.9-fold higher prevalence in eRA patients than controls. However, neither *Aa* nor *P. gingivalis* reached statistical significance. Quantitative PCR displayed similar results where eRA patients displayed 2.9- and 1.8-fold higher prevalence of *Aa* and *P. gingivalis*, respectively, than controls, but neither reached statistical significance (*p* > 0.126).

### 3.3. Potential Oral Bacterial Markers for eRA 

**Figure 3:**
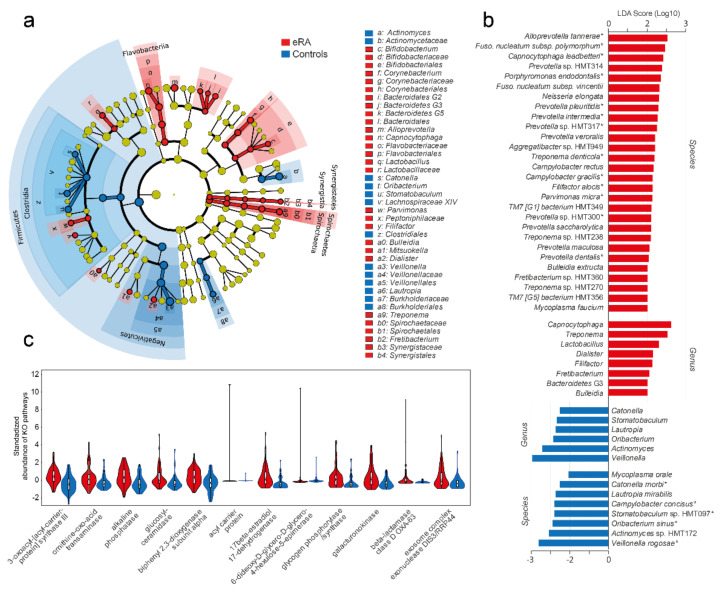
Bacteria (LEfSe) and predicted functions in early onset RA versus healthy subjects. (**a**) Cladogram based on the phylum, class, order, family, genus and species abundances of the 340 bacterial species. (**b**) Bar graph showing species and genera with an LDA score > 2.0. A star (*) by the species indicates that it was detected as influential in the OPLS-DA model too (Figure 2c). (**c**) Violin plots with internal box plots of predicted upregulated microbiota functions in early-onset RA subjects. Using the PICRUSt algorithm, genes and gene families were predicted (KEGG orthologue) based on their 16S rRNA gene sequences. Twelve KEGG orthologues were suggested to be upregulated in early-onset RA. Mann–Whitney U test (Monte Carlo, 1000 permutations, *p* < 0.005) was used to evaluate group differences. All pathways are found in Table S3.

To explore the potential of oral bacteria as biomarkers for eRA, a clade diagram was used to visualize the higher architecture (Figure 3a) and LEfSe to identify potential biomarkers. LDA indicated 28 species (LDA score > 2.0; FDR, 0.05; *p* < 0.007) to be associated with eRA patients (Figure 3b) of which 13 coincided with the top 17 RA associated species identified by OPLS-DA (Figure 2c). LDA identified 8 species to be associated with healthy controls (LDA > 2.0, FDR, 0.05; *p* < 0.007) including all 5 species identified by OPLS-DA (Figure 3b).

### 3.4. Predicted Bacterial Functions in eRA Patients and Healthy Controls

In view of the compositional differences in the oral microbiota of eRA versus healthy controls, we hypothesized that innate microbiota functions differed accordingly. From the PICRUSt algorithm, KEGG orthologues (KO) were predicted based on the 16S rRNA gene sequences and the reference Greengenes genome database. A total of 10,543 KOs were identified, with a significant difference between eRA and healthy controls (PERMANOVA, 9999 permutations, FDR-adjusted, Euclidean distance; *p* = 0.0021). Of the 79 unique KOs that differed between eRA and healthy controls (Mann–Whitney, Monte Carlo permutation *n* = 1000; *p* < 0.005; Table S3), 12 were suggested to be higher in eRA than healthy controls (Figure 3c), whereas 67 were higher in the healthy controls. The KOs increased in eRA were assigned to functions in fatty acid metabolism, ornithine metabolism, glucosylceramidase, sphingolipids, beta-lactamase resistance, biphenyl degradation and 17-beta-estradiol 17-dehydrogenase metabolism. Most of the KOs that were increased in healthy controls were associated with functions in metabolic pathways, microbial metabolism in diverse environments, degradation of aromatic compounds, two-component system, nicotinate and nicotinamide metabolism, ATP-binding cassette transporters, biosynthesis of antibiotics and toluene degradation.

### 3.5. Oral Microbiota in eRA and Periodontal Status

**Figure 4:**
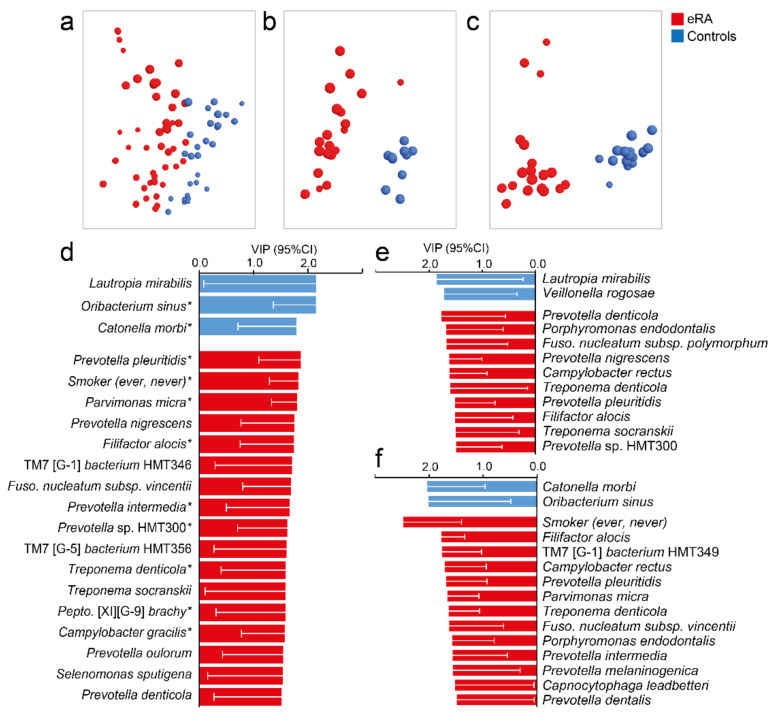
Saliva microbiota pattern by periodontal status in early onset RA versus healthy subjects. (**a**–**c**) Loading plots from OPLS-DA modelling in (**a**) all participants for whom dental data could be obtained, (**b**) in eRA subjects and healthy controls with no sign of periodontal disease, and (**c**) in e RA subjects and healthy controls with PPD of ≥6 mm. (**d**–**f**) Bar graphs showing bacterial species and additional variables influential in the model projections in (**d**–**f**). Data are expressed as Variable In Projection (VIP) values with 95% confidence interval, for (**d**) eRA patients and healthy controls with dental data, (**e**) eRA patients and healthy controls with no sign of periodontal disease and (**f**) eRA patients and healthy controls with a sign of periodontal disease. Red colour represents eRA and blue represents healthy controls. For a full list of VIP values, see Table S4. A star (*) by the species indicates that it was detected as influential in the OPLS-DA.

Given that several bacterial species that were enriched in eRA patients are known to be associated with periodontal disease, a supervised multivariate OPLS-DA model with species abundances was run in healthy controls and eRA patients for whom dental status could be linked. Species abundances separated the eRA from the healthy controls (Figure 4a) and confirmed most of the top 20 species identified by OPLS-DA among the 120 participants (Figure 2c) and/or by LDA (LeFSe) (Figure 3b). This included *Prevotella pleuritidis*, *Filifactor alocis*, *Treponema denticola*, *Peptostreptococcaceae* [XI][G-9] *brachy*, *Prevotella* sp. HMT300, *Campylobacter gracilis*, *Prevotella intermedicus* and *Parvimonas micra* in eRA patients and *Lautropia mirabilis* and *Veillonella rogosae* in healthy controls.

Subsequently, we divided the participants into eRA patients and healthy controls with no sign of periodontal disease (Figure 4b) and eRA patients and healthy controls with a sign of periodontal disease (Figure 4c) and ran separate OPLS-DA models. Increased abundance of *Prevotella pleuritidis*, *Porphyromonas endodontalis*, *Filifactor alocis* and *Treponema denticola* were associated with eRA regardless of periodontal status (Figure 4e,f) though with deviating ranking positions. These species also coincided with species found influential among all 61 eRA patients (Figure 2c). Additional influential species among eRA patients without deepened PPD were *Prevotella denticola*, *Prevotella nigrescens*, *Fusobacterium subsp. polymorphum* and *Treponema socranskii* (Figure 4e). Patients with eRA and with 6 mm PPD were also, besides being a present or past smoker, characterized by *Capnocytophaga leadbetteri*, *Prevotella dentalis*, *Prevotella intermedia*, *Prevotella melaninogenica*, *Parvimonas micra*, *Fusobacterium* subsp. *vincentii* and *Saccharibacteria* (TM7) [G-1] *bacterium* HMT349 (Figure 4f). Healthy controls without signs of periodontal disease were characterized by *Lautropia mirabilis* and *Veillonella rogosae* and those with 6 mm PPD by *Catonella morbi* and *Oribacterium sinus* (Figure 4f).

## 4. Discussion

This study compared saliva microbiota profiles in early untreated RA patients and healthy controls by multiplex 16S rRNA gene sequencing. The saliva microbiota in eRA patients was distinctive from that of healthy controls when restricting analyses to those with no clinical sign of any deepened periodontal probing pocket. Further, eRA patients displayed more dissimilar and dispersed microbiota profile and enrichment for periodontitis-associated taxa beyond *P. gingivalis* and *Aa*, including the emerging periodontal pathogen *Filifactor alocis*. Possibly, identified species could be developed into markers for eRA risk assessment.

In the present study, oral microbiota features in patients with early untreated RA were evaluated, whereas most previous studies have targeted patients with established RA [19,39,40]. Several findings in the eRA patients were in accordance with findings in established RA, such as higher species richness and an overall differing taxa profile compared with healthy controls [19]. A lack of different microbial diversity between healthy subjects and RA patients has also been reported [6,39], as well as decreased species richness of the saliva microbiota in other autoimmune diseases, such as Sjögren’s syndrome and Behçet’s disease [41,42]. In general, enrichments of periodontitis-associated species, such as in *Prevotella*, *Alloprevotella*, *Fusobacterium* and *Filifactor* were reported in patients with established RA [19,43,44]. A few studies, mainly recent ones, have targeted early-stage RA and patients with ACPA positive arthralgia but no sign of arthritis, i.e., patients at risk for RA [6,9,18,21]. Some report that periodontal signs are more severe already at early RA/at-risk-stages [6,18], but this was not confirmed in recent Dutch [21] or, though there was a tendency, in the present Swedish population. However, all these studies find support for dysbiotic traits in the oral biofilms, i.e., subgingival, saliva or tongue scrapings in eRA or individuals at risk for RA. Similar to the results in the present study, most candidate species have been linked to periodontal disease. Still, the reported top candidate species differ by study, even for the same type of samples. Some find significant enrichment of *P. gingivalis* in ACPA positive individuals at risk for RA but only at periodontally healthy sites [9], others highlight *Filifactor alocis* [45], or species in the *Prevotella* and *Veillonella* genera [21] and yet others report a significant role of *Aa*, *Filifactor alocis*, *Campylobacter rectus*, *Porphyromonas endodontalis* and *Treponema vincentii* as important bacterial network hubs [18]. Though direct comparisons between these studies and the present study in eRA are difficult since underlying populations and their dental care, sampling types, targeted variable regions or gene site in PCR, sequencing method, bioinformatic criteria, and 16S rRNA reference database differ. However, several periodontitis-related species found in our eRA group overlapped with those reported in the published eRA or ACPA positive individuals at risk for RA, such as enriched *Filifactor alocis* [45], species in the *Prevotella* genus [6] and *Porphyromonas endodontalis.*

We found that *Filifactor alocis* was enriched in the eRA patients, supporting a potential link between *Filifactor alocis* and RA progression. In addition to the close relatedness of *Clostridium* and *Fusobacterium*, in silico analysis of the *Filifactor alocis* genome identified an arginine deiminase pathway involved in arginine metabolism leading to citrulline and ornithine. It has also been reported that the five genes involved in arginine metabolism are highly upregulated during co-infection with *P. gingivalis. Filifactor alocis* has been suggested to be able to convert arginine to ornithine without the citrulline intermediate step potentially contributing to ornithine increase [46]. In this study, the L-ornithine-converting enzyme (L-Ornithine:2-oxo-acid aminotransferase) was predicted to be elevated by 1.5-fold in the microbiota of eRA patients (*p* = 0.00006). Further, a recent study has identified a novel exotoxin, in the RTX toxin superfamily of cytolysins and cytotoxins isolated from *Filifactor alocis* [47]. Thus, further studies on *Filifactor alocis* in RA development may elucidate nouvelle roles for the species alone or in combination with other bacterial species.

*Fusobacterium* species, previously associated with both RA [19,48] and periodontal disease [49], were confirmed to be more prevalent in eRA patients. *Fusobacterium nucleatum subsp. vincentii* was 2.2-fold more common, and *Fusobacterium nucleatum subsp. polymorphum* was more abundant in eRA patients than in healthy controls. Additional bacteria associated with eRA and known to be linked to periodontal disease were in the *Prevotella* and *Fretibacterium* genera. Whether these species are causally associated with RA or are a biomarker for periodontal disease linked to RA remains to be evaluated, as is the increased abundance and prevalence of *Campylobacter* species [50]. Nonetheless, the results of this study are in line with our previous finding that periodontal disease is associated with prospective RA development [51] and other published studies on early RA [9,18].

Mechanistically, PPAD produced by *P. gingivalis*, has been shown to catalyze citrullination of both bacterial and host proteins, potentially contributing to the production of ACPA, although recently disputed [12,15,52]. We did not detect statistically significant enrichment of *P. gingivalis* abundance in the saliva of eRA patients, though there was a several-fold higher prevalence among these patients by both 16S rDNA sequencing and qPCR. Notably, 10 different *Porphyromonas* phylotypes were identified in the saliva microbiota, of which *Porphyromonas endodontalis* was significantly enhanced in eRA patients. *P. endodontalis* does not harbour the *PAD* gene [53] but has been linked to periodontitis [54] and another inflammatory disease, e.g., Sjögren’s syndrome [41].

Additionally, *Aa* leukotoxin, an acylated protein toxin and a major virulence factor in periodontitis [55], drives the hypercitrullination of RA autoantigens in human neutrophils, suggesting a link between this species and RA [17]. Accordingly, leukotoxin-producing *Aa* strains are reported as more prevalent in RA, and antibodies against leukotoxin A are associated with HLA-shared epitope and ACPA and RF positivity [17]. In the current study, *Aa* was detected in five participants, all of whom were RA patients, which, although not statistically significant, may support a link between *Aa* and RA.

Prediction of potential functions in the oral microbiota revealed that the eRA group was characterized by fewer KEGG-derived KOs though their bacteria species richness was comparably high, whereas the healthy control group was characterized by 5 times as many KOs but less species richness than in the eRA group. This suggests that several species in the eRA group had overlapping functions, which is supported by the enrichment of several species in some genera, such as in *Prevotella* and *Treponema*, in the eRA microbiota. Elevated levels of bacterial glucosylceramidase (K01201, GBA1) were indicated in the eRA-microbiota, which is potentially linked to sphingolipid synthesis in, e.g., *Prevotella* and *Porphyromonas*, which may provoke a broad systemic response, including apoptosis and inflammation [56].

It is well established in observational studies that patients with established RA have worse periodontal status than healthy controls [57], and there is support in our previous study that a worse periodontal status precedes the debut of RA [51]. The question that emerged when evaluating the present bacterial strains enriched in eRA patients over age and sex-matched controls is if the finding is merely driven by a worse periodontal status. Therefore, we performed sensitivity analyses in subgroups of eRA patients and controls that were free of clinical signs of deepened periodontal pockets and found that the differing microbiota profile persisted. This supports the role of oral bacteria in triggering RA-related processes. However, we based our assumption on PPD merely and cannot exclude the eRA had signs of early inflammation, such as bleeding gums.

The strength of the present study was that well-characterised eRA patients could be studied before any disease modifying anti-rheumatic treatment was introduced and that information on PPD could be linked for 65% of the participants and that groups of eRA and controls free of or with deepened PPD could be defined and used for sensitivity analyses. The limitations include that 16S rRNA gene segments from multiplex next-generation sequencing limits taxonomic resolution. Although we used the curated eHOMD oral database, there was still a risk of misclassification of homologous species, but this was unlikely to have a significant overall impact as the species of interest were present in several participants and represented by many reads. Additional aspects to be considered when interpreting these results are the limited abilities to adjust for potential genetic factors, diet, or stress, which may have an effect on the oral microbiota composition, whereas no RA-patient had started any treatment with an anti-rheumatic drug beyond corticosteroid treatment, which was found unrelated to the microbiota composition. A further potential limitation is the cross-sectional case-control study design, but patients diagnosed with RA are subjected to treatment which often includes, e.g., methotrexate, sulphasalazine, leflunomide or hydroxychloroquine, and even also biological DMARDs and often in parallel with the treatment of any oral condition to reduce any inflammation locus. These actions are likely to alter the oral microbiota and, therefore, hampered a longitudinal study design. The present study also included eRA patients who had been treated for a short time with corticosteroids, even though, within the limits of this study, there was no microbiota change linked to corticosteroid treatment.

## 5. Conclusions

The microbiota comparison between patients with eRA and healthy controls displayed distinct diversity differences and notable enrichments of *Filifactor alocis*, *Porphyromonas endodontalis*, *Prevotella pleuritidis* and *Treponema denticola* in the eRA patients, a finding that was confirmed regardless of if periodontal signs were present or not. We propose by the results from this study that oral infections with certain bacteria alone or in inter-bacterial symbiosis or competition in the ecosystem could be an initiator in a genetically predisposed individual. These findings support the previously suggested distinct association between periodontal disease and RA. The results also suggest that the use of oral bacteria as potential biomarkers for RA development should be further evaluated, including the role and mechanisms of *Porphyromonas endodontalis* and *Filifactor alocis* in RA induction. 

## Figures and Tables

**Table 1:** Participant characteristics for the early onset RA group and healthy controls sampled for saliva microbiota evaluation.

	Healthy Controls *n* = 59	Early RA *n* = 61	*p*-Value
Women, *n* (%)	44 (74.6)	46 (75.4)	0.916
Age, mean (SD), years	57 (13)	58 (15)	0.667
Symptoms before diagnosis, mean (SEM), months	-	7.8 (0.7)	-
Rheumatoid factor+, *n* %	nt	45 (73.8)	-
ACPA+, *n* %	nt	43 (70.5)	-
DAS28, mean (SEM)	nt	4.5 (0.2)	
Prednisolone, *n* (%)	0	16 (26.2)	
**Smoking**			<0.001
never smoker, *n* (%)	55 (93.2)	34 (55.7)	
former-smoker (%)	3 (5.1)	22 (36.1)	
current smoker, *n* (%)	1 (1.7)	5 (8.2)	
**Dental status**, ***n*** (%)	30	48	
number of teeth ^1^	26.8 (1.4)	25.5 (1.6)	0.091
number of teeth with PPD ≥ 6 mm, mean (SD) ^1^	1.3 (1.3)	2.6 (4.4)	0.089

nt = not tested, DAS28 = disease activity score [24]. ^1^ adjusted for sex and age at the dental examination.

## Data Availability

The 16s rRNA sequencing data set is available on Figshare via the links 10.6084/m9.figshare.11365274.v1 and 10.6084/m9.figshare.11365301.v1. The datasets generated and/or analysed during the current study are not publicly available due Swedish legislation but anonymised data may be available from the corresponding author on reasonable request and adequate ethical approval. Other data are available upon reasonable request and acquired mandatory ethical approvals.

## Supplementary Materials

The following are available online at https://www.mdpi.com/article/10.3390/microorganisms9081657/s1, Table S1: Bacterial taxa in saliva from early RA (eRA) patients versus healthy control subjects, Table S2: Bacterial species discriminating early RA from healthy control subjects in OPLS-DA regression modelling, Table S3: Predicted microbiome function in early RA and healthy controls, Table S4: Bacterial species discriminating early RA (eRA) patients from healthy control subjects taking periodontal status into account.
